# Body Composition and Physical Fitness Affect Central Hemodynamics in Young Children

**DOI:** 10.3389/fped.2021.750398

**Published:** 2021-10-27

**Authors:** Sabrina Köchli, Arne Deiseroth, Christoph Hauser, Lukas Streese, Arno Schmidt-Trucksäss, Oliver Faude, Henner Hanssen

**Affiliations:** Department of Sport, Exercise and Health, Medical Faculty, University of Basel, Basel, Switzerland

**Keywords:** body mass index, physical fitness, central hemodynamics, children, early vascular aging

## Abstract

**Objective:** Central hemodynamics are related to cardiovascular (CV) outcomes in adults, but associations with childhood CV risk remain unclear. The study aimed to investigate the association of obesity, physical activity, and fitness with parameters of central pulse wave reflection in young prepubertal children.

**Methods:** In this cross-sectional study, 1,324 primary school children (aged 7.2 ± 0.4 years) were screened for parameters of pulse wave reflection such as augmentation index (AIx), central pulse pressure (CPP), body mass index (BMI), and cardiorespiratory fitness (CRF) by standardized procedures for children.

**Results:** The mean AIx and AIx@75 were 22.2 ± 7.7 and 29.2 ± 9.2%, respectively. With each unit increase in BMI, AIx [−0.226 (−0.328; −0.125)%] and AIx@75 [−0.444(−0.660; −0.229)%] decreased, whereas peak forward pulse wave increased (*p* < 0.001). Increasing BMI was associated with higher CPP, but did not remain significant after adjustment for CRF and heart rate. One unit increase in CRF was associated with lower AIx@75 [−0.509(−0.844; −0.173)%, *p* = 0.003] and lower reflection magnitude [RM: −0.559 (−0.890; −0.227), *p* = 0.001], independent of body weight and height. Girls had significantly higher AIx, AIx@75, peak backward pulse wave, and RM compared with boys.

**Conclusion:** Childhood obesity was associated with higher CPP but lower augmentation of the reflected pulse wave in children. Assessment of central blood pressures appears to be a valuable asset to childhood CV risk screening. The validity of augmentation indices during childhood development and the association with early vascular aging in children need to be verified in long-term follow-up studies. Physical activity and fitness have the potential to improve vascular hemodynamics in susceptible children and, thus, counteract vascular aging.

**Trial registry: ClinicalTrials.gov:** Exercise and Arterial Modulation in Youth. **Identifier:** NCT02853747; URL: https://clinicaltrials.gov/ct2/show/NCT02853747.

## Introduction

Structural and functional changes in large arteries, commonly assessed by arterial stiffness, are related to the pathogenesis of cardiovascular (CV) disease. Aortic augmentation index (AIx), measured by pulse wave analysis (PWA), is a non-invasive and validated vascular biomarker to assess large arterial stiffness (1, 2). AIx values depend on the relative contribution of forward and reflected pulse waves to blood pressure. In addition, the characteristic pulse wave can be separated into forward (Pf) and backward wave (Pb) to calculate reflection magnitude (RM). A previous meta-analysis demonstrated that central hemodynamic wave reflections are the main determinants of cardiovascular (CV) events and all-cause mortality (3). Obesity is one of modern day's main risk factors for the development of CV disease with an increasing global prevalence of physical inactivity and unhealthy dietary intake (4, 5). In adults, body fat has been associated with higher central and peripheral AIx (6). In contrast, body mass index (BMI) was negatively correlated with AIx in a healthy population (6). Fernberg et al. found that young Swedish adults with obesity and low physical fitness had higher AIx compared with peers without obesity (7). A meta-analysis from randomized controlled trials showed that high-intensity aerobic exercise has merits to improve arterial stiffness and wave reflection in adults (8). Childhood obesity and elevated blood pressure (BP) seem to play a key role in mediating a deleterious CV outcome later in life (9, 10). However, the mechanisms of early subclinical hemodynamic changes from childhood until adulthood are still poorly understood. A few studies measured central hemodynamic parameters in children. We recently demonstrated that childhood obesity, hypertension, and low physical fitness were associated with higher pulse wave velocity (11). There is evidence that obesity and elevated BP are predominantly associated with a higher Pf and Pb in children and adolescents (12, 13). Children with obesity have been reported to present with lower AIx compared with normal weight children (12). A negative correlation has been reported between body mass index (BMI) and central AIx (14). However, some studies found no association of childhood obesity with AIx (15, 16). Physical activity and fitness have the potential to counteract the development of childhood obesity and vascular impairments (17, 18). The association of physical activity and fitness with AIx, Pf, Pb, and RM and central BP have never been investigated in young children. Our study, for the first time, aimed to examine the association of body composition and cardiorespiratory fitness (CRF) with central hemodynamic parameters in young children.

## Method

### Study Design and Participants

Data were collected from the large-scale, cross-sectional EXAMIN YOUTH study (19). Inclusion criteria were that children between the ages of 6 and 8 years are allowed to participate in physical education lessons and had a letter of agreement from their parents. Children had to remain fasted in the morning of the medical test day. Physical fitness assessments took place on-site in regular physical education lessons. The study was designed according to the Guidelines for Good Clinical Practice of the Declaration of Helsinki, (20) and ethical approval was obtained by the Ethics Committee of the University of Basel (EKBB, Basel, No. 258/12). The manuscript conforms to The Strengthening the Reporting of Observational Studies in Epidemiology (STROBE) Guidelines.

### Measurements

#### Central Hemodynamics

PWA was performed using the oscillometric Mobil-O-Graph Monitor, which has been validated in adults (I.E.M. GmbH, Germany) (21–23). The oscillometric assessment of hemodynamic parameters is strongly associated with the conventional tonometric method in adults (*r* = 0.71, *p* < 0.001 for AIx) (21). The measurements were performed in a calm environment in a sitting position. Appropriate small-sized cuffs for children were placed on the left upper arm. After a 5-min resting period, calibration was conducted based on systolic BP. Afterward, PWA was performed and immediately checked for quality and inaccurate data. At least two valid measurements were used to calculate the mean and standard deviation of AIx, AIx corrected for heart rate (AIx@75), Pb, Pf, RM (defined as the ratio of the amplitude of the Pb to that of the Pf), central systolic BP (CSBP), central diastolic BP (CDBP), and central pulse pressure (CPP).

#### Anthropometrics

Anthropometric parameters were assessed in light sport clothes and no shoes. Body height was measured with a wall-mounted stadiometer (Seca 206; Seca, Basel, Switzerland). To assess body weight and body fat, an electrical impedance device (InBody 170 Biospace device; InBody Co., Seoul, Korea) was used. BMI was calculated as body weight in kilograms divided by the square of height. Children were classified in clinical relevant groups according to cut off points for BMI incorporating age and sex (24). Children with a BMI over the 85th percentile were categorized as overweight and over the 95th percentile as children with obesity.

#### Blood Pressure

BP was measured with an automated oscillograph (Oscillomate 9002, CAS Medical Systems, Branford, CT, USA), similar models of which have been validated in children (25, 26). After a rest of 5 min in a sitting position, five BP measurements were performed. The mean of the three measurements with the smallest variation was taken for further analysis. According to the population-based German KiGGs study (27) and the 2016 European guidelines (28), children were categorized in systolic and diastolic BP groups defined as normal BP (<90th percentile), high-normal BP (>90th percentile), and hypertension (>95th percentile).

#### Cardiorespiratory Fitness, Physical Activity, and Screen Time

The 20-m shuttle run test was performed to assess CRF. The validated and well-established 20-m shuttle run is an indicator of CRF and maximal endurance exercise capacity (29, 30). After a short warm-up, children had to run continuously between two 20-m lines back and forth as long as possible. The running speed was synchronized with acoustic bleep signals. The running velocity was increased every stage of 1 min by 0.5 km/h from an initial speed of 8 km/h. The test score was achieved if a child failed to cross the lines twice in a row. The number of stages reached (with an accuracy of 0.5 stages) was used for further analysis. Parents were asked to complete a questionnaire about the physical activity level and screen time of their children, based on our previous studies (31, 32). Physical activity was defined by time spent in vigorous physical activity (min/day). Screen time was assessed by questions included watching TV, playing computer or video games, and playing on a smartphone (min/day).

### Statistical Analysis

The residuals were analyzed by using Tukey–Anscombe plots and normal QQ plots to assess variance homogeneity and normality. Multilinear regression analysis was performed to analyze central hemodynamic parameters (AIx, AIx@75, Pf, Pb, RM, CSBP, CDBP, CPP) with body composition and physical activity/fitness. Different models were fitted to adjust for age and sex as well as body weight, height, and shuttle run. Univariate analysis of variance (ANOVA) was used to compare differences of central hemodynamic parameters between BMI and BP categories. A two-tailed *p*-value of 0.05 indicates statistical significance, and 95% confidence intervals were presented for measures of effect of uncertainty. Normal density curve was presented as a histogram of continuous variables of AIx and AIx@75. Statistical analyses were performed with an up-to-date version of Stata 15 (StataCorp LP, College Station, TX, USA).

## Results

**Figure 1:**
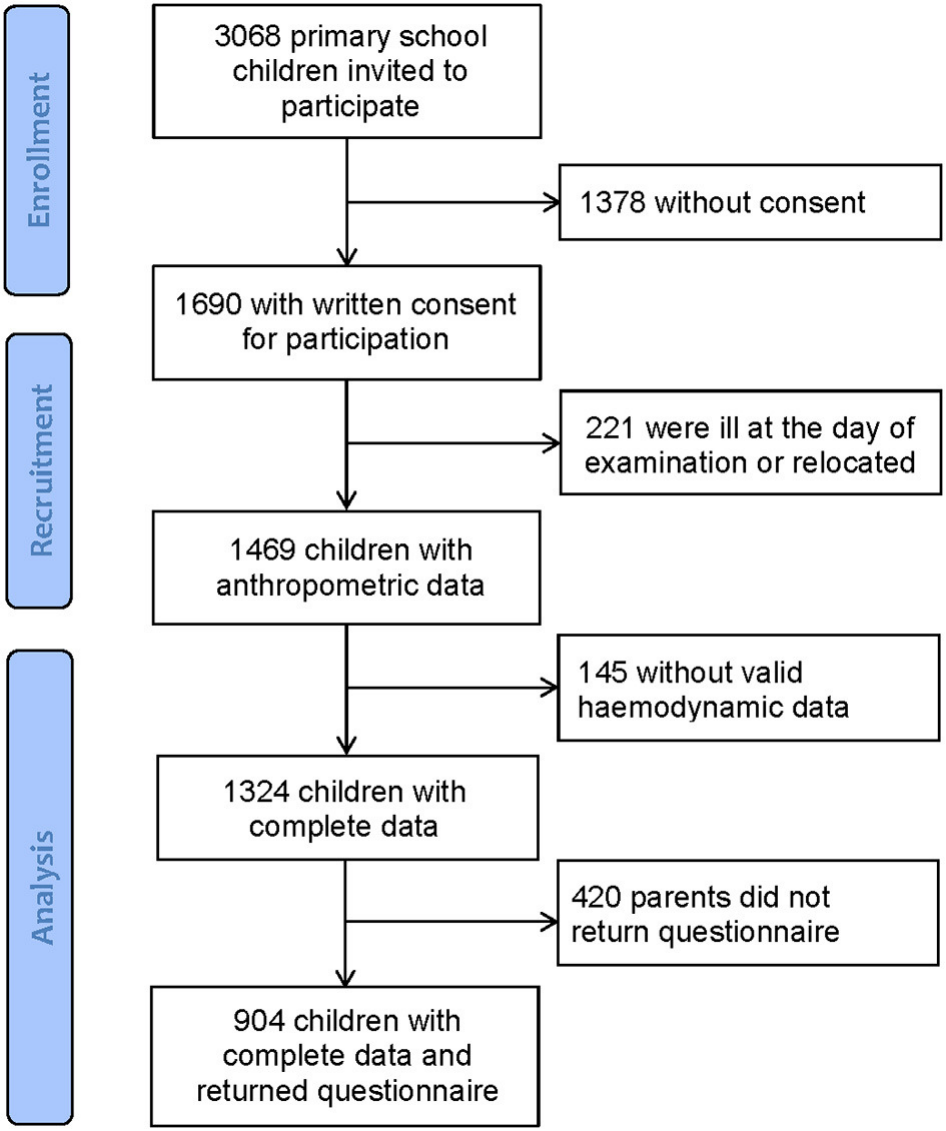
Flow diagram.

Population characteristics are shown in Table 1. Overall, 3,068 school children received an invitation to take part in this large-scale cross-sectional study. From 1,690 children with written consent from their parents, 366 children were ill at the day of examination or had insufficient quality of hemodynamic data. In total, 1,324 children completed all measurements. Parents (420) did not return the questionnaire, leaving 904 children with complete data including questionnaires. Figure 1 shows the flow diagram. According to the BMI categories, 87% (*n* = 1,154) were of normal weight, 10% (*n* = 126) were overweight, and 3% (*n* = 44) were children with obesity.

**Table 1:** Population characteristics of the study.

**Parameter**	**Total**	***n***	**Boys**	***n***	**Girls**	***n***	***p***
	**Mean ± SD**		**Mean ± SD**		**Mean ± SD**		
Age	7.2 ± 0.4	1324	7.2 ± 0.4	652	7.2 ± 0.3	672	0.164
Height (cm)	124.5 ± 5.6	1324	124.7 ± 5.3	652	124.2 ± 5.9	672	0.113
Weight (kg)	24.7 ± 4.8	1324	24.8 ± 4.5	652	24.6 ± 5.0	672	0.390
BMI (kg/m^2^)	15.4 ± 2.2	1324	15.9 ± 2.1	652	15.8 ± 2.3	672	0.695
Percentage body fat (%)	15.4 ± 7.7	1324	13.7 ± 7.0	652	17.0 ± 8.0	672	<0.001
Heart rate (bpm)	85.7 ± 10.4	1324	85.1 ± 10.2	652	86.2 ± 10.5	672	0.060
Systolic BP (mmHg)	103.8 ± 7.8	1324	103.8 ± 7.6	652	103.8 ± 7.9	672	0.998
Diastolic BP (mmHg)	64.1 ± 6.9	1324	64.1 ± 6.9	652	64.1 ± 6.9	672	0.829
Mean arterial BP (mmHg)	77.3 ± 6.5	1324	77.4 ± 6.6	652	77.3 ± 6.7	672	0.880
Central systolic BP (mmHg)	91.6 ± 8.0	1324	91.3 ± 8.0	652	91.9 ± 8.0	672	0.161
Central diastolic BP (mmHg)	61.6 ± 6.7	1324	61.6 ± 6.7	652	61.6 ± 6.6	672	0.880
Central pulse pressure (mmHg)	30.0 ± 6.0	1324	29.7 ± 6.1	652	30.3 ± 5.9	672	0.082
AIx (%)	22.2 ± 7.7	1324	19.5 ± 7.4	652	24.7 ± 7.1	672	<0.001
AIx@75 (%)	29.2 ± 9.2	1324	26.1 ± 9.2	652	32.5 ± 8.2	672	<0.001
Pf (mmHg)	20.4 ± 3.6	1324	20.5 ± 3.7	652	20.4 ± 3.5	672	0.597
Pb (mmHg)	12.2 ± 3.0	1324	12.0 ± 3.1	652	12.4 ± 2.9	672	0.016
RM	59.5 ± 8.8	1324	58.4 ± 9.7	652	60.6 ± 7.7	672	<0.001
20-m Shuttle Run (stages)	3.8 ± 1.5	1324	4.0 ± 1.6	652	3.4 ± 1.3	672	<0.001
Vigorous physical activity (min/day)	67.8 ± 50.5	766	78.1 ± 54.1	307	57.0 ± 44.1	315	<0.001
Screen time (min/day)	55.5 ± 60.4	904	58.1 ± 56.5	356	52.7 ± 64.0	377	0.180

*BMI, body mass index; BP, blood pressure; AIx, Augmentation index; AIx@75, Augmentation index normalized for a heart rate of 75 bpm; Pf, peak forward pressure; Pb, peak backward pressure; RM, reflection magnitude; PWV, pulse wave velocity; SD, standard deviation*.

**Figure 2:**
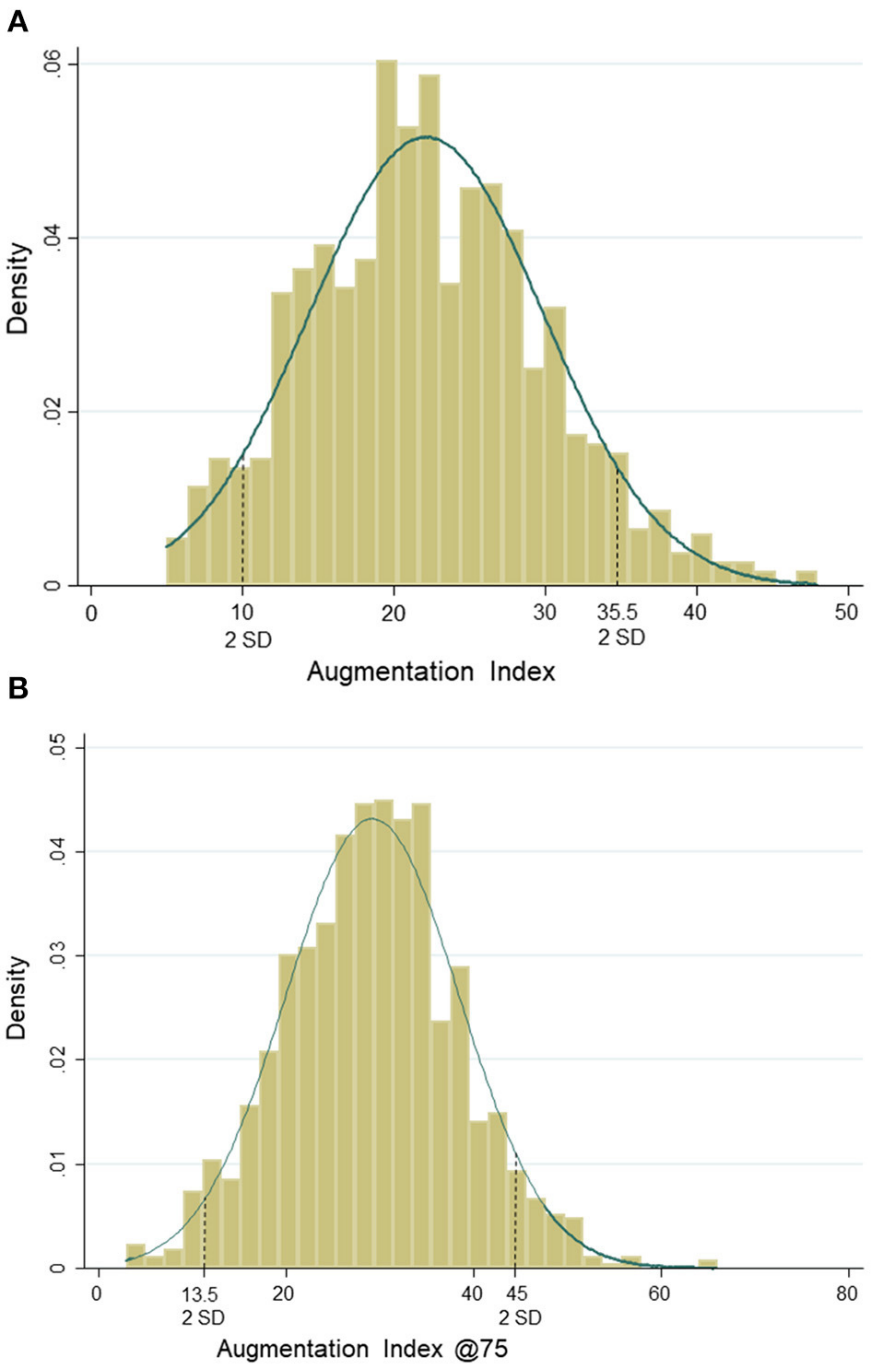
**(A)** Normal distribution of augmentation index. **(B)** Normal distribution of augmentation index@75.

Based on peripheral systolic BP, 77% (*n* = 1,023) of the children had a normal BP, 9% (*n* = 123) were classified as children with a high-normal BP, and 13% (*n* = 178) were with hypertension. Based on peripheral diastolic BP, 78% (*n* = 1,030) were categorized as children with a normal BP, 8% (*n* = 112) as high normal, and 14% (*n* = 182) were children with hypertension. In total, 7% (*n* = 90) of the children were categorized as children with both systolic and diastolic hypertension. In our study, 89% of children were Caucasian. Girls had significantly higher AIx, AIx@75, Pb, RM, percentage body fat, and lower CRF and vigorous physical activity compared with boys. Mean AIx and AIx@75 were 22.2 ± 7.7 and 29.2 ± 9.2, respectively. Normal density curves of AIx and AIx@75 are presented in Figures 2A,B.

### Regression Analysis

The regression analysis for parameters of pulse wave reflection is shown in Table 2. Lower AIx (*p* < 0.001) and AIx@75 (*p* < 0.001) were found per unit increase in BMI, independent of CRF and heart rate. Higher BMI was associated with higher Pf (*p* < 0.001) and lower RM (*p* < 0.001), even after adjustment for confounders. One-unit increase in body height was independently related to lower AIx, AIx@75, Pb, and RM (*p* < 0.001). After adjustment for body height, body weight was associated with lower AIx@75 (*p* = 0.019), higher Pf (*p* < 0.001), and Pb (*p* = 0.028). One-unit increase in percentage body fat was associated with lower AIx (*p* < 0.022) and higher Pf (*p* < 0.001). After adjustment for body height and weight, higher CRF was associated with lower AIx@75 (*p* = 0.003) and RM (*p* < 0.001).

**Table 2:** Regression analysis for the association of body composition, physical activity, and fitness with parameters of pulse wave reflection.

**Parameter**	**Model**	**Aix** **(% change per unit)**		**AIx@75** **(% change per unit)**		**Pf** **(mmHg change per unit)**		**Pb** **(mmHg change per unit)**		**RM** **(change per unit)**	
		**B (95% CI)**	***p***	**B (95% CI)**	***p***	**B (95% CI)**	***p***	**B (95% CI)**	***p***	**B (95% CI)**	***p***
Body height (cm)	1 2	−0.292 (−0.364; −0.221) −0.226 (−0.328; −0.125)	<0.001 <0.001	−0.347 (−0.433; −0.262) −0.256 (−0.377; −0.135)	<0.001 <0.001	0.035 (−0.002; −0.071) −0.040 (−0.091; −0.011)	0.061 0.123	−0.073 (−0.102; −0.043) −0.109 (−0.151; −0.068)	<0.001 <0.001	−0.459 (−0.543; −0.374) −0.423 (−0.542; −0.304)	<0.001 <0.001
Body weight (kg)	1 3	−0.285 (−0.368; −0.202) −0.113 (−0.235; 0.008)	<0.001 0.068	−0.331 (−0.430; −0.232) −0.174 (−0.319;−0.029)	<0.001 0.019	0.089 (0.047; 0.130) 0.124 (0.063; 0.185)	<0.001 <0.001	−0.023 (−0.057; 0.012) 0.056 (0.006; 0.106)	0.199 0.028	−0.371 (−0.470; −0.272) −0.084 (−0.227; 0.059)	<0.001 0.249
BMI (kg/m^2^)	1 4	−0.398 (−0.578; −0.217) −0.425 (−0.617; −0.234)	<0.001 <0.001	−0.444 (−0.660; −0.229) −0.563 (−0.791; −0.335)	<0.001 <0.001	0.191 (0.102; 0.280) 0.198 (0.104; 0.293)	<0.001 <0.001	0.032 (−0.042; 0.106) 0.008 (−0.071; 0.086)	0.398 0.849	−0.402 (−0.619; −0.185) −0.535 (−0.765; −0.305)	<0.001 <0.001
Percentage body fat (%)	1 4	−0.064 (−0.117;−0.011) −0.069 (−0.128; −0.010)	0.018 0.022	−0.017 (−0.081; 0.047) −0.048 (−0.119; 0.022)	0.602 0.178	0.050 (0.024; 0.076) 0.054 (0.025; 0.083)	<0.001 <0.001	0.029 (0.007; 0.050) 0.023 (−0.001; 0.047)	0.010 0.061	−0.010 (−0.074; 0.055) −0.046 (−0.117; 0.025)	0.770 0.205
20-m shuttle run (stages)	1 5	0.094 (−0.177; 0.365) −0.104 (−0.385; 0.178)	0.496 0.470	−0.240 (−0.564; 0.083) −0.509 (−0.844; −0.173)	0.146 0.003	−0.068 (−0.202; 0.065) 0.039 (−0.103; 0.180)	0.317 0.592	−0.111 (−0.221; −0.5E−3 −0.098 (−0.214; 0.019)	0.049 0.100	−0.314 (−0.640; 0.011) −0.559 (−0.890; −0.227)	0.058 0.001
Vigorous physical activity (min/day)	1 5	−0.012 (−0.022; −0.001) −0.009 (−0.019; −0.001)	0.029 0.075	−0.010 (−0.022; 0.002) −0.007 (−0.019; 0.005)	0.111 0.233	0.001 (−0.004; 0.006) 0.6E−3 (−0.005; 0.006)	0.628 0.827	−0.001 (−0.006; 0.003) −0.001 (−0.005; 0.003)	0.584 0.641	−0.013 (−0.025; 0.3E−3) −0.010 (−0.022; 0.003)	0.055 0.126
Screen time (min/day)	1 5	0.007 (−0.9E−3; 0.015) 0.008 (0.2E−3; 0.016)	0.085 0.044	0.014 (0.005; 0.024) 0.016 (0.006; 0.025)	0.003 0.001	0.002 (−0.002; 0.006) 0.7E−3 (−0.003; 0.005)	0.368 0.733	0.004 (0.7E−3; 0.007) 0.003 (0.1E−3; 0.007)	0.018 0.041	0.013 (0.004; 0.023) 0.014 (0.005; 0.023)	0.007 0.004

*Model 1, adjusted for age and sex; Model 2, model 1 plus adjusted for body weight and shuttle run; Model 3, model 1 plus adjusted for body height and shuttle run; Model 4, model 1 plus adjusted for shuttle run; Model 5, model 1 plus adjusted for body height and body weight*.

*BMI, body mass index; AIx, augmentation index; AIx@75, augmentation index normalized for a heart rate of 75 bpm; Pf, peak forward pressure; Pb, peak backward pressure; RM, reflection magnitude; CI, confidence interval*.

Linear associations of AIx (*p* = 0.044), AIx@75 (*p* = 0.001), Pb (*p* = 0.041), and RM (*p* = 0.004) with screen time were found. Table 3 shows regression analysis for the association of body composition, physical activity, and fitness with central BP and CPP. One-unit increase in BMI was independently associated with higher CSBP (*p* < 0.001) and CDBP (*p* < 0.001). Higher CPP was associated with BMI (*p* < 0.010), but not independent of CRF and heart rate. Percentage body fat was associated with higher CSBP (*p* < 0.001) and CDBP (*p* < 0.001) and CPP (*p* = 0.003). One-unit increase in shuttle run (stages) was associated with lower CSBP and CDBP (*p* < 0.001), but the results did not remain significant after adjustment for body height, body weight, and heart rate.

**Table 3:** Regression analysis for the association of body composition, physical activity, and fitness with central blood pressure and pulse pressure.

**Parameter**	**Model**	**CSBP (mmHg change per unit)**		**CDBP (mmHg change per unit)**		**CPP (mmHg change per unit)**	
		**B (95% CI)**	***p***	**B (95% CI)**	***p***	**B (95% CI)**	***p***
Body height (cm)	1 2	0.247 (0.168; 0.327) −0.179 (−0.284; −0.073)	<0.001 0.001	0.319 (0.254; 384) 0.005 (−0.081; 0.091)	<0.001 0.910	−0.071 (−0.132; −0.010) −0.184 (−0.270; −0.099)	0.022 <0.001
Body weight (kg)	1 3	0.579 (0.493; 0.666) 0.721 (0.595; 0.848)	<0.001 <0.001	0.541 (0.470; 0.613) 0.552 (0.449; 0.654)	<0.001 <0.001	0.040 (−0.030; 0.109) 0.172 (0.069;0.274)	0.264 0.001
BMI (kg/m^2^)	1 4	1.273 (1.087; 1.460) 1.249 (0.1.052; 1.445)	<0.001 <0.001	1.084 (0.929; 1.239) 1.093 (0.931; 1.260)	<0.001 <0.001	0.194 (0.044; 0.344) 0.159 (−0.4E−3; 0.319)	0.011 0.051
Percentage body fat (%)	1 4	0.375 (0.320; 0.430) 0.370 (0.310; 0.430)	<0.001 <0.001	0.299 (0.253; 345) 0.300 (0.248; 0.349)	<0.001 <0.001	0.078 (0.034; 0.121) 0.073 (0.024; 0.122)	0.001 0.003
20-m shuttle run (stages)	1 5	−0.824 (−1.117; −0.530) −0.122 (−0.417; 0.172)	<0.001 0.415	−0.618 (−0.863; −0.372) 0.007 (−0.232; 0.246)	<0.001 0.958	−0.213 (−0.437; 0.012) −0.133 (−0.371; 0.105)	0.063 0.274
Vigorous physical activity (min/day)	1 5	0.008 (−0.003; 0.019) 0.003 (−0.008; 0.014)	0.150 0.564	0.009 (−0.001; 0.018) 0.003 (−0.005; 0.012)	0.080 0.451	−0.3E−3 (−0.009; 0.008) −0.4E−3 (−0.009; 0.008)	0.942 0.934
Screen time (min/day)	1 5	0.014 (0.005; 0.022) 0.007 (−0.002; 0.015)	0.002 0.133	0.007 (0.5E−8; 0.015) 0.001 (−0.006; 0.008)	0.050 0.737	0.007 (0.6E−4; 0.013) 0.006 (−0.001; 0.012)	0.048 0.100

*Model 1, adjusted for age and sex; Model 2, model 1 plus adjusted for body weight, shuttle run, and heart rate; Model 3, model 1 plus adjusted for body height, shuttle run, and heart rate; Model 4, model 1 plus adjusted for shuttle run and heart rate; Model 5, model 1 plus adjusted for body height, body weight, and heart rate*.

*BMI, body mass index; CSBP, central systolic blood pressure; CDBP, central diastolic blood pressure; CPP, central pulse pressure; CI, confidence interval*.

### Group Differences Across Clinical Categories

Central hemodynamic parameters of pulse wave reflection in relation to clinical categories of BMI and BP are shown in Supplementary Table 1. BMI categories were not associated with AIx and AIx@75. Children with overweight and obesity had higher Pf compared with peers with normal weight (*p* = 0.001). According to systolic BP categories, children with high-normal BP and hypertension showed higher AIx@75 compared with children with normal BP (*p* < 0.001). Pf (*p* = 0.002) and Pb (*p* = 0.001) increased according to increasing systolic BP categories. Young children with diastolic hypertension showed higher AIx (*p* = 0.043) and AIx@75 (*p* < 0.001) compared with children with high-normal and normal BP.

## Discussion

This study is the first to analyze the association of body composition and physical fitness with central hemodynamics in a large cohort of young prepubertal children. Higher BMI was associated with lower AIx, AIx@75, and RM. Higher body weight and body fat were independently related to higher CSBP, CDBP, and CPP. Physical fitness was associated with favorably lower AIx@75, RM, Pb, CSBP, and CDBP.

In line with the results of our large cohort of young children, a previous smaller-sized study found that a lower AIx and a higher Pf in children with obesity compared with normal weight peers (12). Indeed, findings on the association of BMI with central pulse wave velocity as a marker of arterial stiffness are inconsistent (11, 16, 33, 34). Studies in adolescents found no association of childhood obesity with AIx and AIx@75 (15, 16). Childhood growth and development during puberty and adolescence may be responsible for the loss of associations of body composition with parameters of pulse wave reflection. Indeed, a premature decline of arterial stiffness has been suggested due to accelerated growth and early onset of puberty (35).

We have previously analyzed the association of BMI with central pulse wave velocity in the same population of primary school children (11). One unit increase in BMI was independently related to a higher pulse wave velocity in this cohort of children [0.027 (0.010; 0.034), *p* < 0.001]. In contrast to these previous findings, we have now found an inverse association of BMI with AIx, which appears conflicting and remains to be clarified in future studies.

With respect to the AIx and AIx@75, body height and heart rate need to be discussed as potential influencing factors for lower augmentation in children with higher BMI. We demonstrated that body weight is associated with AIx, but not independent of body height. In our cohort, children with obesity were significantly taller than children with normal weight (data not shown). Body height seems to be a determinant for a lower AIx in children with a higher body weight. Higher Pf and Pb were related to body weight, independent of body height. A lower AIx@75 was independently associated with higher body weight. Moreover, our findings indicate that not only body height but also heart rate seems to be a key factor for determining AIx, but less so for AIx@75 as this is a set value at a heart rate of 75/min. In our cohort of young children, similar values of AIx and AIx@75 were found compared with values in older adults (36). It may mainly be explained by short stature of children and less so by higher heart rate (mean 85.7 ± 10.4 bpm) in children at a young age. Potential inaccuracy of the oscillometric device in children may add to this phenomenon (37).

Our study is the first to assess AIx and AIx@75 in a large population-based unselected cohort of 6–8 year-old children, offering reliable normal values for young Caucasian children (mean AIx: 22.2 ± 7.7% and AIx@75: 29.2 ± 9.2%). We have recently demonstrated that low physical fitness and sedentary behavior seem to play a key role in the development of micro- and macrovascular impairments in young children (11). These results are in line with our current findings in pulse wave reflection. Higher CRF and lower physical inactivity (screen time) were associated with a favorably lower AIx@75 and RM. A higher AIx and Pf were found per unit increase of screen time. Independent of body composition and physical activity level, elevated peripheral BP affected alterations in central pulse wave parameters. Previous studies showed that elevated BP is associated with a higher Pf and Pb in children and adolescents (12, 13). Similar to these results, we found that children with systolic hypertension had higher AIx@75, Pf, and Pb. A few studies investigated the association of early life conditions with central BP and CPP. In our study, higher body weight and percentage body fat were independently associated with higher CSBP, CDBP, and CPP. We found that CRF was associated with lower CSBP and CDBP, but not independent of body weight and height. Girls had a higher AIx, AIx@75, Pb, RM, and percentage body fat but lower CRF and vigorous physical activity compared with boys. Similar to our findings, a recent study demonstrated that AIx@75 was higher in girls compared with that in boys before puberty (38). The authors argued that early gender differences in aortic geometry and growth are potential reasons for these findings (38). Besides childhood growth, low CRF seems to be a determinant for the development of higher AIx, AIx@75, Pb, and RM in girls. Our results suggest that improving CRF, often accompanied by weight loss, has the potential to ameliorate large-artery hemodynamics in young children.

Some of the potential pathophysiological mechanisms need to be discussed. Central hemodynamic impairments and obesity-related inflammation are mediated through oxidative stress conditions (39). Oxidative stress plays a major role in nitric oxide (NO) bioavailability, a main determinant of vascular tone regulation and vasodilation. Lower levels of NO are, therefore, likely to affect pulse wave reflection. Low CRF and physical inactivity are characterized by reduced mitochondrial capacity and increased oxidative stress, and regular physical exercise has the potential to improve oxidative conditions and NO bioavailability (40).

This study has some limitations that need to be addressed. The study is designed as a cross-sectional investigation and does not examine temporal development of the associations. A long-term follow-up is warranted to confirm causal associations between lifestyle-related risk factors and the development and progression of vascular properties. Only three percent of children in our study were children with obesity. Central hemodynamics may need to be investigated in an obesity-enriched cohort. Our study was performed in a predominant Caucasian population. Future studies have to prove our findings in other ethnic populations. Moreover, it needs to be stressed that measurements of central hemodynamics have been validated in adults but not in children. Recent reports indicated that oscillometric as well as tonometric devices may not be accurate indices of invasively measured AIx in children (37). From the KidCoreBP study, it also appears that MobilOgraph may overestimate peripheral and central BP (41). However, potential inaccuracy of central BP measurement would be systematic in nature, and associations with risk would remain representative in a population-based approach. Our data suggests that using AIx in children should be handled with caution. Finally, a potential selection bias between participants and non-participants cannot be excluded. One strength of the study is the large number of participants. During childhood, age-related changes in vascular structure and function occur rapidly. Growth and developmental effects are minimized by the large sample size and limited age range of children aged 6–8 years.

## Conclusion

In summary, our results demonstrate that higher BMI in young pre-pubertal children is associated with lower augmentation of the reflected pulse wave. Compared with pulse wave velocity, AIx is more susceptible to factors such as body height and heart rate, which vary greatly throughout inter-individual childhood development. Our results offer normal values of central hemodynamics for a foremost Caucasian population of 6–8-year-old children. Nonetheless, it has to be stated that the use of AIx in childhood needs to be handled with caution. Assessment of central BP appears to be a more valuable asset to childhood CV risk screening. The clinical relevance and predictive value of augmentation indices during childhood development and the association with early vascular aging and CV risk in adulthood remain to be verified in long-term follow-up studies. Based on the available literature and our previous findings, pulse wave velocity seems a more robust marker to be included in childhood CV risk screening. Our results do support recommendations for physical activity programs to be included in childhood primary prevention strategies to improve childhood vascular health and counteract early vascular aging in susceptible children.

## Data Availability Statement

The raw data supporting the conclusions of this article will be made available by the authors, without undue reservation.

## Ethics Statement

This study was reviewed and approved by Ethics Committee of North-West Switzerland: No.258/12. Written informed consent to participate in this study was provided by the participants' legal guardian/next of kin.

## Author Contributions

SK performed the recruitment, data collection and analysis, and prepared the manuscript draft. AD, CH, LS, AS-T, and OF revised the manuscript and approved the final draft. HH conceptualized the study, supported the data analysis, revised the manuscript, and approved the final draft. All authors contributed to the article and approved the submitted version.

## Conflict of Interest

The authors declare that the research was conducted in the absence of any commercial or financial relationships that could be construed as a potential conflict of interest. The handling Editor declared a past collaboration with several of the authors SK, HH.

## Publisher's Note

All claims expressed in this article are solely those of the authors and do not necessarily represent those of their affiliated organizations, or those of the publisher, the editors and the reviewers. Any product that may be evaluated in this article, or claim that may be made by its manufacturer, is not guaranteed or endorsed by the publisher.

## Abbreviations

Aix: augmentation index
AIx@75: augmentation index for a heart rate of 75 bpm
BMI: body mass index
BP: blood pressure
CDBP: central diastolic blood pressure
CPP: central pulse pressure
CRF: cardiorespiratory fitness
CSBP: central systolic blood pressure
CV: cardiovascular
NO: nitric oxide:
Pb: peak backward pulse wave
Pf: peak forward pulse wave
RM: reflection magnitude.
