# Supplementary material for: Body Composition and Physical Fitness Affect Central Hemodynamics in Young Children
Source: Front Pediatr. 2021 Oct 27;9:750398. doi: 10.3389/fped.2021.750398 (PMC8578851; doi:10.3389/fped.2021.750398)
Supplement: Supplementary file 1 [file Table_1.DOCX]

| **Supplement Table 1.** Central pulse wave reflection parameters in relation to clinical categories of body mass index and blood pressure. | | | | | | | | | |  |  |
| --- | --- | --- | --- | --- | --- | --- | --- | --- | --- | --- | --- |
| **Parameter** | **n** | **AIx (%)**  **Mean (95% CI)** | **p** | **AIx@75 (%)**  **Mean (95% CI)** | **p** | **Pf (mmHg)**  **Mean (95% CI)** | **p** | **Pb (mmHg)**  **Mean (95% CI)** | **p** | **RM**  **Mean (95% CI)** | **p** |
| BMI  Normal weight  Overweight  Obese | 1154  126  44 | 22.3 (21.9;22.8)  20.9 (19.6;22.3)  21.0 (18.7;23.3) | 0.085 | 29.4 (28.8;29.9)  27.8 (26.8;29.4)  28.9 (26.2;31.6) | 0.200 | 20.3 (20.1;20.5)  21.4 (20.7;22.0)  22.0 (20.9;23.0) | 0.001 | 12.2 (12.0;12.3)  12.5 (11.9;13.0)  13.0 (12.1;13.9) | 0.120 | 59.3 (58.7;59.9)  57.3 (55.6;59.6)  58.2 (55.1;61.4) | 0.252 |
| Systolic BP  Normotensive  High-normal  Hypertensive | 1023  123  178 | 21.9 (21.4;22.4)  22.8 (21.5;22.4)  23.2 (22.1;24.4) | 0.065 | 28.4 (27.9;29.0)  30.8 (29.2;32.5)  32.6 (31.3;34.0) | <0.001 | 20.3 (20.1;20.5)  20.5 (19.9;21.2)  21.3 (20.8;21.8) | 0.002 | 12.1 (11.9;12.3)  12.4 (11.9;13.0)  12.9 (12.5;13.4) | 0.001 | 58.8 (58.2;59.5)  59.4 (57.5;61.2)  60.7 (59.0;62.3) | 0.123 |
| Diastolic BP  Normotensive  High-normal  Hypertensive | 1030  112  182 | 21.9 (21.4;22.3)  23.1 (21.7;24.5)  23.2 (22.1;24.3) | 0.043 | 28.4 (27.8;29.0)  31.3 (29.6;33.0)  32.4 (31.1;33.8) | <0.001 | 20.5 (20.3;20.7)  20.2 (19.5;20.8)  20.2 (19.7;20.8) | 0.446 | 12.2 (12.0;12.4)  12.3 (11.7;12.9)  12.3 (11.9;12.7) | 0.871 | 58.6 (57.9;59.2)  61.5 (59.6;63.4)  60.4 (58.9;62.0) | 0.004 |

P value across lowest and highest category (univariate analysis of variance)

BMI, body mass index; BP, blood pressure; AIx, Augmentation index; AIx@75, Augmentation index normalized for a heart rate of 75 bpm; Pf, peak forward pressure; Pb, peak backward pressure; RM, reflection magnitude; CI, confidence interval
